# Paracrine Factors from Irradiated Peripheral Blood Mononuclear Cells Improve Skin Regeneration and Angiogenesis in a Porcine Burn Model

**DOI:** 10.1038/srep25168

**Published:** 2016-04-29

**Authors:** Stefan Hacker, Rainer Mittermayr, Stefanie Nickl, Thomas Haider, Diana Lebherz-Eichinger, Lucian Beer, Andreas Mitterbauer, Harald Leiss, Matthias Zimmermann, Thomas Schweiger, Claudia Keibl, Helmut Hofbauer, Christian Gabriel, Mariann Pavone-Gyöngyösi, Heinz Redl, Erwin Tschachler, Michael Mildner, Hendrik Jan Ankersmit

**Affiliations:** 1 Division of Plastic and Reconstructive Surgery, Medical University of Vienna, Waehringer Guertel 18-20, 1090 Vienna, Austria; 2 Christian Doppler Laboratory for Cardiac and Thoracic Diagnosis and Regeneration, Waehringer Guertel 18-20, 1090 Vienna, Austria; 3 Ludwig Boltzmann Institute for Experimental and Clinical Traumatology, AUVA Research Center, Donaueschingenstraße 13, 1200 Vienna, Austria; 4 Department of Trauma Surgery, Medical University of Vienna, Waehringer Guertel 18-20, 1090 Vienna, Austria; 5 Division of Rheumatology, Medical University of Vienna, Waehringer Guertel 18-20, 1090 Vienna, Austria; 6 Red Cross Blood Transfusion Service of Upper Austria, Krankenhausstraße 7, 4017 Linz, Austria; 7 Division of Cardiology, Medical University of Vienna, Waehringer Guertel 18-20, 1090 Vienna, Austria; 8 Department of Dermatology, Medical University of Vienna, Waehringer Guertel 18-20, 1090 Vienna, Austria; 9 Division of Thoracic Surgery, Medical University of Vienna, Waehringer Guertel 18-20, 1090 Vienna, Austria

## Abstract

Burn wounds pose a serious threat to patients and often require surgical treatment. Skin grafting aims to achieve wound closure but requires a well-vascularized wound bed. The secretome of peripheral blood mononuclear cells (PBMCs) has been shown to improve wound healing and angiogenesis. We hypothesized that topical application of the PBMC secretome would improve the quality of regenerating skin, increase angiogenesis, and reduce scar formation after burn injury and skin grafting in a porcine model. Full-thickness burn injuries were created on the back of female pigs. Necrotic areas were excised and the wounds were covered with split-thickness mesh skin grafts. Wounds were treated repeatedly with either the secretome of cultured PBMCs (Sec^PBMC^), apoptotic PBMCs (Apo-Sec^PBMC^), or controls. The wounds treated with Apo-Sec^PBMC^ had an increased epidermal thickness, higher number of rete ridges, and more advanced epidermal differentiation than controls. The samples treated with Apo-Sec^PBMC^ had a two-fold increase in CD31+ cells, indicating more angiogenesis. These data suggest that the repeated application of Apo-Sec^PBMC^ significantly improves epidermal thickness, angiogenesis, and skin quality in a porcine model of burn injury and skin grafting.

Extensive burn wounds represent a serious trauma to affected patients and require a well-orchestrated interdisciplinary effort by the treating physicians. Over the last few decades, early excision and skin grafting has emerged as the treatment of choice for deep partial-thickness and full-thickness burns, leading to a significant reduction in mortality^1^,^2^. Autologous split-thickness skin grafts are the gold standard for permanent closure of burn wounds. Skin grafts are usually expanded using mesh grafting, transplantation of preformed skin stamps according to the modified Meek technique, micrografts, or other techniques in order to overcome the discrepancy between relatively small areas of healthy donor skin and extensive areas of burned skin^3^,^4^,^5^. The Meek technique is named after its inventor and describes the use of standardized 3 × 3 mm micrografts that are produced by a commercially available cutting machine. Due to the great expansion ratio, this method has been used for the coverage of large burns (>60% Total body surface area)^4^. Current experimental approaches aim to further improve the expansion ratio of harvested skin transplants^6^. However, higher expansion rates lead to greater open wound areas left to heal by secondary intention between the transplanted skin. Early permanent coverage of these areas is crucial in the treatment of extensive burns, as any delays may lead to hypothermia, protein and fluid loss, and an increased risk of infection^7^.

Successful treatment of open wounds with split-thickness skin grafts is dependent on the vascular supply of the transplant and early adequate angiogenesis^8^. Thus far, various attempts to improve vessel ingrowth and to increase the take rate of split-thickness skin grafts have yielded inconclusive results^9^,^10^. Under ideal conditions, split-thickness skin grafting is a reliable treatment option with a high success rate. However, several factors may lead to graft failure, such as infections, reduced angiogenesis, oedema, insufficient preparation of the wound bed, or motion of the graft bed^11^. Wound healing after skin grafting requires the integration of complex events, including cell proliferation, cell migration, extracellular matrix deposition, and tissue remodelling. Many of these events are directed by cytokines, chemokines, and growth factors expressed at the different stages of the regeneration process^12^,^13^,^14^. Several authors have aimed to improve wound healing by adding external soluble factors, stem cells, or progenitor cells to acute or chronic wounds. The addition of stem cells, genetically modified cutaneous cells, recombinant proteins, or paracrine factors obtained from *in vitro* cell cultures has led to encouraging results^15^,^16^,^17^,^18^. In particular, paracrine factors have shown beneficial effects on wound healing mechanisms, as they act as pro-angiogenic and anti-apoptotic modulators, increasing cell proliferation and migration^18^,^19^,^20^,^21^,^22^. The idea of therapeutically utilizing the complex mixture of factors secreted by cultured cells originates from stem cell research. Gnecchi *et al*. showed that the conditioned medium of stem cells, rather than the cells themselves, is responsible for many of the observed beneficial effects of stem cell therapy^23^. The repertoire of paracrine factors secreted by cells under *in vitro* culture conditions has been shown to comprise numerous cytoprotective and angiogenic factors^20^,^24^,^25^. This conditioned cell culture medium contains a plethora of soluble factors, called the “secretome”, and can be harvested by standard cell culture methods. The components of the secretome of cultured peripheral blood mononuclear cells (PBMCs) used in this study were examined in several previous studies by our group^26^,^27^. It comprises released proteins, lipids, and extracellular vesicles (microparticles and exosomes) and its composition can be actively altered by exposing the cells to external stressors like ionizing radiation (IR)^27^,^28^.

Despite recent developments, the isolation of stem cells and subsequent harvesting of their secretomes remains a costly and time-consuming process. In contrast, PBMCs are an easily obtainable cell source and represent an interesting alternative to stem cells in their capacity to act as “bioreactors” for the production of paracrine factors^29^,^30^. In a previous study we found that PBMCs release a multitude of paracrine cytokines and growth factors, even under standard cell culture conditions^26^. In 2005, Thum *et al*. reported that a large fraction of the stem cells used for therapy after myocardial infarction undergo apoptosis, and that many of the observed beneficial effects may be due to these “dying stem cells”^31^. Perotti *et al*. found that lethally irradiated cord blood mononuclear cells have therapeutic effects when administered in critical limb ischaemia^32^. Based on these observations, we decided to reproduce this effect using PBMCs. We applied gamma irradiation to induce apoptosis in PBMC cultures. After irradiation, the secretion pattern of the apoptotic PBMCs changed significantly and led to even higher production of cytokines and chemokines, including large amounts of IL-8 and VEGF^26^. We previously analysed the effects of the secretome derived from apoptotic PBMCs in functional *in vivo* and *in vitro* experiments. We demonstrated that it significantly accelerated wound healing in a mouse model of full-thickness skin wounds *in vivo* and led to enhanced migration of human primary keratinocytes and fibroblasts and increased proliferation of human primary microvascular endothelial cells *in vitro*^18^. In addition to the observed effects on cutaneous wound healing, the secretome derived from apoptotic PBMCs has been shown to possess a therapeutic capacity in animal models of myocardial infarction. The functional outcome was markedly improved, mainly due to cytoprotective effects on cardiomyocytes^26^,^33^,^34^. In an experimental animal model of autoimmune myocarditis, the secretome of PBMCs was able to effectively attenuate myocardial inflammation, suggesting an anti-inflammatory effect^35^. The secretome of PBMCs was also successfully used to mitigate the pathophysiological processes of secondary damage after spinal cord injury in an animal model^36^. The selective blocking of single growth factors or cytokines did not abrogate the observed effects of the PBMC secretome *in vitro*, indicating that these effects are caused by the mixture of paracrine factors rather than a single pathway or factor^33^.

Based on these results, we analysed the effect of PBMC-derived secretomes on wound healing in a previously established porcine model of full-thickness burn and 24-hour delayed excision and split-thickness skin grafting that closely resembles the clinical situation of acute burn wound treatment in humans^7^,^37^.

## Results

### Treatment with secretomes does not induce systemic inflammation or wound infection

**Figure 1:**
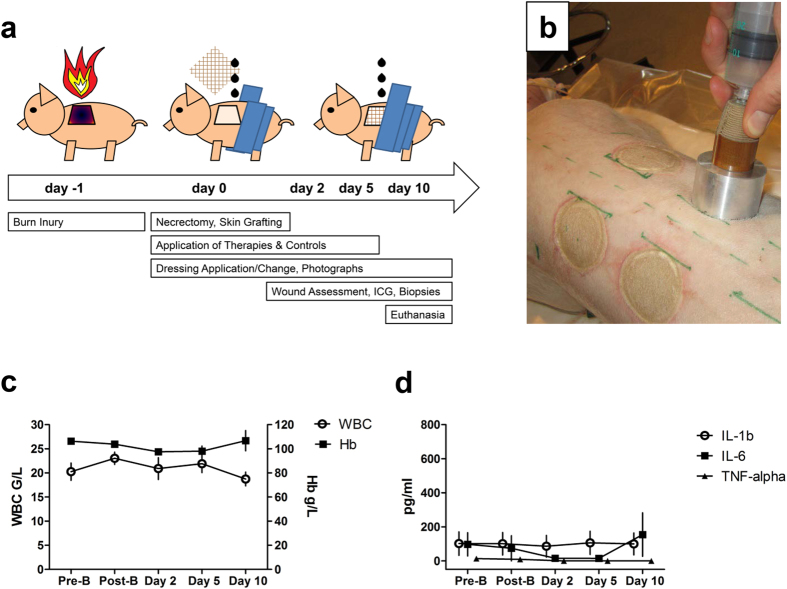
Creation of burn injuries and topical treatment with PBMC secretomes was well tolerated. (**a**) Study timeline. (**b**) A custom-made device was used to create burn wounds on the back of female pigs prior to necrectomy and skin-grafting. (**c**,**d**) Routine laboratory parameters showed no signs of infection or anaemia during the study period. Error bars indicate standard error of the mean (SEM). n = 6.

To exclude potential systemic effects of applying the secretomes, we performed clinical check-ups and regular blood draws before and after the burn injury, as well as on days 2, 5 and 10. All animals recovered quickly from the surgical interventions and general anaesthesia and all wounds remained without clinical signs of infection throughout the study period. The split-thickness skin graft donor sites healed completely without complications within the 10-day study period. The clinical evaluations and laboratory parameters (haemoglobin [Hb], white blood count [WBC], IL-6, IL-1b, TNF-alpha) did not reveal systemic signs of infection or blood loss (Fig. 1c,d).

### Planimetric measurements show a trend towards less wound contraction after secretome treatment

**Figure 2:**
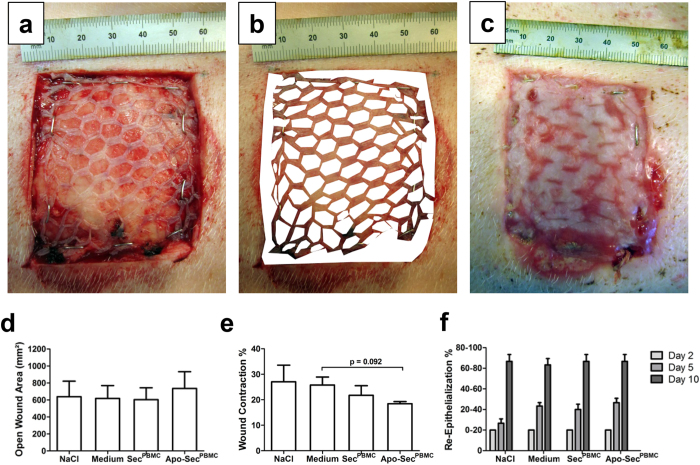
Planimetric evaluation of wounds. Standardized photographs of the wounds were evaluated regarding the open wound area and the ratio of wound contraction using ImageJ software on postoperative day 0 (**a**,**b**) and day 10 (**c**). The white areas indicate the open wound immediately after surgery. (**d**) The extent of the open wound area was comparable between all groups on day 10. (**e**) The wounds treated with Apo-Sec^PBMC^ had a trend towards reduced wound contraction rate on day 10 compared to the medium control group. (**f**) Re-epithelialization rates on days 2, 5 and 10 are shown. Error bars indicate standard error of the mean (SEM). n = 6

To evaluate the macroscopic features of the healing phase, photographs taken at the time of the last dressing change were analysed by two blinded observers in regards to closure of the open wound area, wound contraction, and take rate of the skin grafts (Fig. 2a–c). On postoperative day 10, the remaining open wound area was approximately 20% of the initial wound area, with no significant differences between the groups (Fig. 2d). To exclude increased wound closure due to excessive wound contraction, we also measured the rate of wound contraction after 10 days. We found a trend towards less wound contraction in the fields treated with either secretome from living PBMCs (21.8% ± 9.2; Sec^PBMC^) or secretome from apoptotic PBMCs (18.5% ± 2.0; Apo-Sec^PBMC^) compared to the medium (25.8% ± 7.6) or NaCl control (27.1% ± 16.0) (Fig. 2e).

### Clinical wound evaluation and re-epithelialization

In order to mimic the clinical evaluation process used by many surgeons, we utilized a standardized semi-quantitative wound assessment protocol. All wounds were macroscopically assessed according to our wound assessment scheme on the day of surgery and during dressing changes. We found macroscopically comparable results for all wounds at each time point in regards to graft dislocation, graft adherence, fibrin deposition, and granulation tissue (data not shown). No signs of local infection were observed. We found a trend towards faster macroscopic re-epithelialization on postoperative day 5 in wounds treated with Apo-Sec^PBMC^ compared to the NaCl control (P = 0.052). Similar differences were observed between Sec^PBMC^ and the NaCl control. The medium control had a value comparable to the secretome-treated wounds. We found no significant difference on days 2 or 10 (Fig. 2f).

### Secretome treatment has beneficial effects on epidermal regeneration and the epidermal-dermal junction

**Figure 3:**
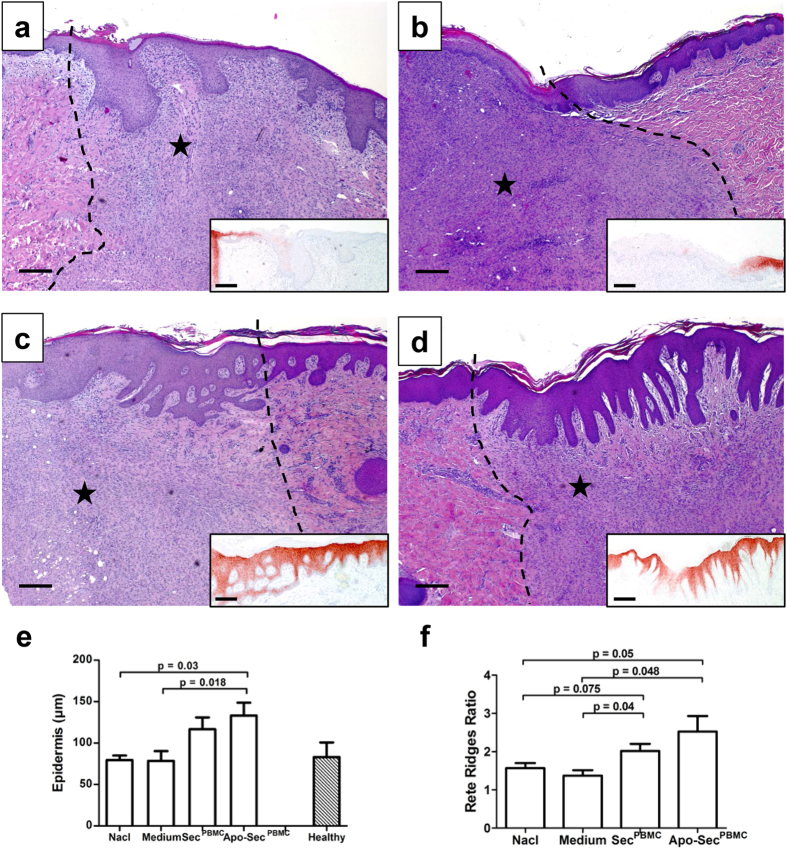
Secretome treatment improves skin quality and epidermal differentiation. Representative H&E staining of the wound edges taken from areas treated with NaCl (**a**), medium (**b**), Sec^PBMC^ (**c**), and Apo-Sec^PBMC^ (**d**). The small inserted sections show the corresponding stainings for the epidermal differentiation marker keratin-10. A progressed epidermal differentiation was observed after treatment with Sec^PBMC^ and Apo-Sec^PBMC^ compared to the control groups. The asterisk (*) indicates the wounded side; the other side shows the healthy, unburned skin. 100× magnification, scale bar: 100 μm. (**e**) The epidermal thickness was markedly increased in the Apo-Sec^PBMC^ group. (**f**) The development of rete ridges – as indicated by a higher ratio between the length of the inner and outer epidermal border – was significantly increased in wounds treated with either Sec^PBMC^ or Apo-Sec^PBMC^ compared to NaCl and medium controls. Error bars indicate SEM. n = 6. Healthy skin: n = 4.

Because fast and stable closure of the interstices between transplanted skin patches is crucial for complete and successful wound healing after skin grafting, we aimed to determine the impact of the PBMC secretome on the quality and degree of epidermal regeneration. The histological characteristics of wounds were quantified on standard haematoxylin and eosin (H&E) cross-sections from biopsies taken on postoperative day 10 (Fig. 3a–d). We found a markedly increased mean epidermal thickness in wounds treated with either Sec^PBMC^ (116.7 μm ± 34.7) or Apo-Sec^PBMC^ (133.2 μm ± 37.6) compared to the medium (78.3 μm ± 29.2) and NaCl groups (79.3 μm ± 13.7). Healthy, unwounded skin had a mean epidermal thickness of 82.9 μm ± 35.7 (Fig. 3e). Rete ridges are epidermal protrusions into the dermal layer and render the epidermal-dermal junction more stable against shear stress. Therefore, we sought to evaluate the rete ridges in standard H&E cross-sections on day 10. The number and quality of rete ridges was improved after repeated application of Sec^PBMC^ or Apo-Sec^PBMC^ compared to the medium or NaCl groups, indicating better stability of the epidermal-dermal junction (Supplementary Fig. S1). In order to compare the length of rete ridges, the ratio between the length of the inner and outer border of the epidermal zone was calculated. Wounds treated with either Apo-Sec^PBMC^ (2.53 ± 1.00; P = 0.05 vs. NaCl and P = 0.048 vs. medium) or Sec^PBMC^ (2.02 ± 0.45; P = 0.075 vs. NaCl and P = 0.04 vs. medium) had a higher ratio than the medium (1.38 ± 0.34) and NaCl control (1.57 ± 0.32) (Fig. 3f).

### Epidermal differentiation is improved after treatment with PBMC secretomes

To evaluate the differentiation of the newly formed epidermal layer, we performed immunohistochemical staining for the late differentiation marker keratin-10 (Fig. 3a–d). Images were taken of the wound margins in order to compare the pre-existing epidermis to the re-epithelialized areas. The differentiation of the newly formed epidermis was markedly progressed in the wounds treated with Apo-Sec^PBMC^. The pre-existing and newly formed epidermis had minimal differences. A similar effect was observed in wounds treated with Sec^PBMC^. However, in the medium and NaCl control wounds, keratin-10 staining was minimal, indicating enhanced regeneration of the epidermal layer over the wound beds after application of the PBMC secretomes.

### Angiogenesis is strongly induced after application of the apoptotic PBMC secretome on day 5

**Figure 4:**
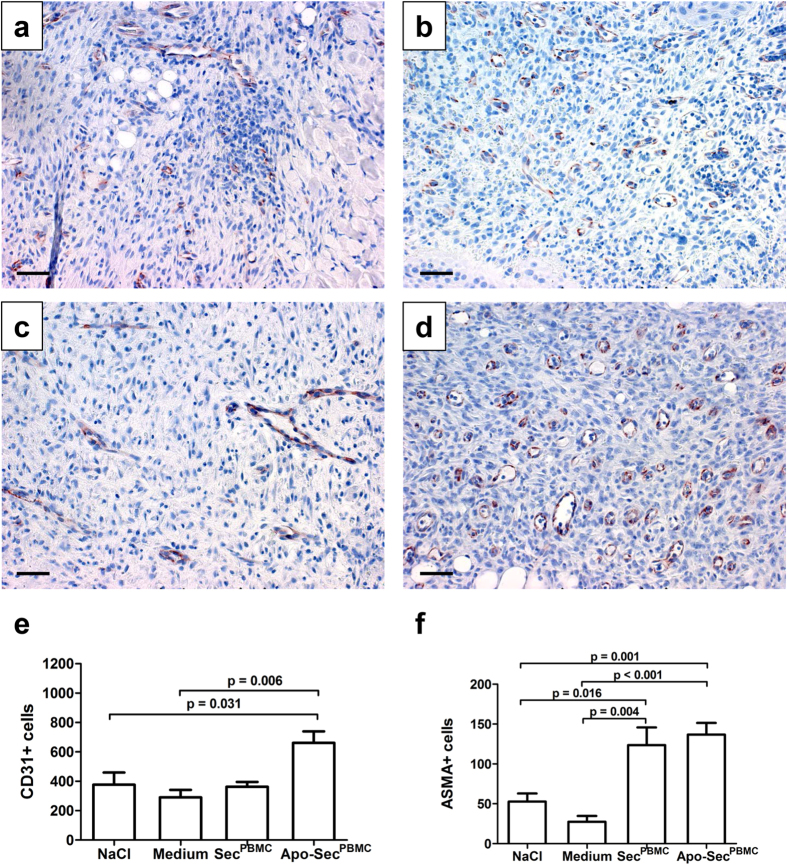
Increased numbers of CD31+ and ASMA cells were observed in wounds treated with PBMC secretomes. Punch biopsy sections taken on day 5 were stained for the angiogenesis marker CD31. Representative samples of the NaCl (**a**), medium (**b**), Sec^PBMC^ (**c**) and Apo-Sec^PBMC^ (**d**) treated wounds are shown. 200× magnification, scale bar: 50 μm. The quantification of CD31+ cells was performed on four randomly selected sections per wound. The numbers correspond to the total amount of cells over four sections. (**e**) Treatment with Apo-Sec^PBMC^ led to a significant two-fold increase in CD31+ cells compared to the control groups. (**f**) Mature blood vessels (ASMA+ cells) were more frequent in the wounds treated with both Sec^PBMC^ and Apo- Sec^PBMC^ compared to the control groups, respectively. Error bars indicate SEM. n = 6.

To investigate the capacity of Sec^PBMC^ and Apo-Sec^PBMC^ to induce angiogenesis *in vivo*, we harvested punch biopsies at the corner of the wounds. We found a strong increase in CD31+ cells in the wounds treated with Apo-Sec^PBMC^ (Fig. 4a–d); the number of CD31+ cells was almost twice as high as in the other groups (Fig. 4e). To support these findings, we performed an additional staining for the mature blood vessel marker alpha smooth muscle Actin (ASMA) and found a significant increase in ASMA+ cell numbers in Apo-Sec^PBMC^-treated wounds compared to the control groups. A similar effect was observed in the wounds treated with Sec^PBMC^ (Fig. 4f and Supplementary Fig. S2). These results indicate a markedly increased ingrowth of blood vessels after topical treatment with the secretome of apoptotic PBMCs. We attempted to confirm these results with dynamic indocyanine green (ICG) measurements but did not find significant differences between the groups (Supplementary Fig. S3). The slope of ICG fluorescence was 2.18 ± 1.18 (NaCl), 2.38 ± 0.82 (medium), 2.42 ± 0.91 (Sec^PBMC^), and 2.42 ± 1.35 (Apo-Sec^PBMC^) and the increase in maximum fluorescence intensity was 23.42% ± 5.75 (NaCl), 26.12% ± 5.18 (medium), 25.26% ± 4.61 (Sec^PBMC^), and 23.81% ± 7.09 (Apo-Sec^PBMC^).

### Mast cell counts are decreased in wounds treated with PBMC secretomes

**Figure 5:**
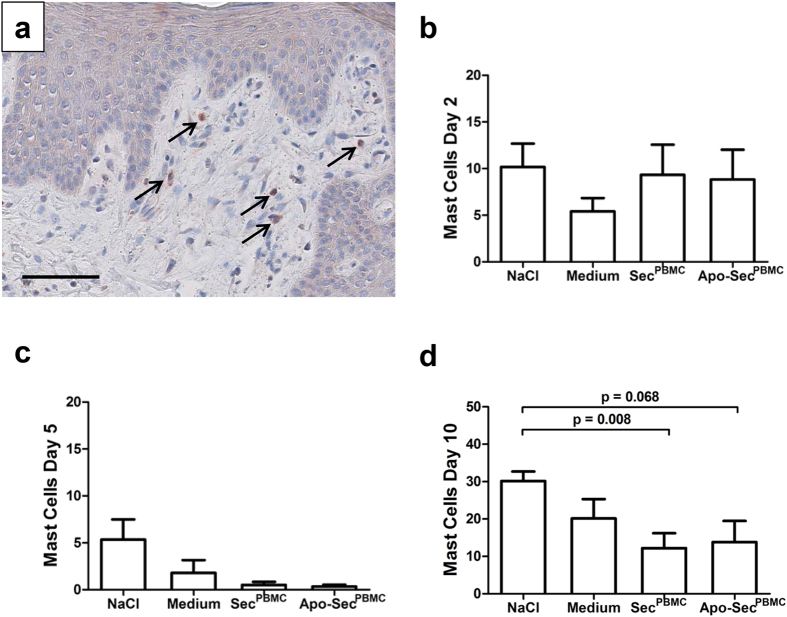
Mast cell counts are reduced after Sec^PBMC^ and Apo-Sec^PBMC^ treatment. Mast cells are found in wounds if derailed scarring occurs. (**a**) Mast cell tryptase-positive cells were found in the superficial layers of the dermis. Arrows indicate mast cells. 400× magnification, scale bar: 50 μm. (**b**) We found no difference in mast cell numbers 2 days after surgery. (**c**) On day 5 we observed a non-significant trend towards fewer mast cells in wounds treated with Sec^PBMC^ or Apo-Sec^PBMC^ compared to the control groups. (**d**) On day 10, this difference was more pronounced. The numbers in the diagrams represent the sum of four randomly chosen sections per wound. Error bars indicate SEM. n = 6.

We also quantified the number of mast cells in wound biopsies over the treatment period. Cells positive for mast cell tryptase were scarce and mostly located in the dermal layer adjacent to the epidermis (Fig. 5a). On day 2, mast cell counts did not differ between treated wounds and the controls (Fig. 5b). However, on day 5 we observed a trend towards diminished mast cell populations in wounds treated with Sec^PBMC^ or Apo-Sec^PBMC^ compared to NaCl controls (Fig. 5c). On day 10 mast cell numbers were significantly different between the fields treated with Sec^PBMC^ and the NaCl controls and showed a strong difference between the Apo-Sec^PBMC^ group and the NaCl group (Fig. 5d).

### Biomechanical properties of wounds

As we were able to observe almost complete wound closure on day 10, we sought to objectively measure the scarring quality of the wounds at the end of the study period using the commercially available Biomechanical Tissue Characterization (BTC-2000™) to assess the biomechanical characteristics of the early scars. We found a trend towards increased laxity of wounds treated with Apo-Sec^PBMC^. We also observed a trend towards better elastic deformation and energy absorption in the Apo-Sec^PBMC^ group. Moreover, scars that developed on Apo-Sec^PBMC^-treated fields also trended towards less stiffness (Table 1).

## Discussion

In this study, we established the feasibility, effectiveness, and safety of topically applying PBMC-derived paracrine factors during burn wound healing *in vivo*. We used a previously described porcine model of full-thickness burns with subsequent necrectomy and split-thickness skin grafting to investigate the effects of Sec^PBMC^ and Apo-Sec^PBMC^ in a scenario closely related to the clinical situation in humans^7^,^37^. We found increased rates of angiogenesis and better epidermal differentiation in wounds treated with Apo-Sec^PBMC^.

Autologous skin grafting has been used by surgeons to treat burn wounds for centuries^38^. Prolonged time to wound closure may result in unfavourable results, such as hypertrophic scarring, contracture, or wound infections^39^,^40^,^41^,^42^. Due to current expansion techniques, such as mesh-graft or Meek, large burn wounds are not entirely covered by autologous skin after surgery but rather by a web of intact, transplanted skin with interspersed open wound areas^3^. Several treatment options, such as the use of skin substitutes or the application of different cell types, including stem cells, have been utilized to improve wound healing after burn injuries^43^,^44^. An interesting alternative to the transplantation of cells is the use of paracrine factors. Previous results with cell-free approaches have been promising and shown improved healing times and scar quality after local application of growth factors^22^,^45^,^46^.

Unlike the complex isolation and cultivation of stem cells and progenitor cells, the acquisition of PBMCs is fast and simple. In a previous study, we characterized the composition of secretomes derived from living (Sec^PBMC^) and irradiated, apoptotic (Apo-Sec^PBMC^) cultured PBMCs, finding an array of pro-angiogenic, cytoprotective, and proliferation factors released into the culture medium over a period of 24 hours. However, the composition and function of the secretome was significantly altered after induction of apoptosis by IR, leading to a higher regenerative capacity^27^,^33^. The application of this mixture of paracrine factors attenuated the immune response and restored functional capacity after induced acute myocardial infarction in rats^34^. Furthermore, these PBMC-derived secretomes exhibited regenerative potential in a murine wound healing model *in vivo*, with strong proliferative and pro-angiogenic effects on cutaneous wounds after topical application^18^. The immunomodulatory effects of Apo-Sec^PBMC^ have been shown in a porcine model of myocardial remodelling. Local administration of Apo-Sec^PBMC^ led to silencing of genes involved in apoptosis and inflammation^47^. Burn wounds are prone to the occurrence of secondary damage due to excessive inflammation and immunomodulatory treatments were able to improve wound healing after burn injury^48^. In order to better mimic the clinical setting in humans, we used a porcine model of full-thickness burn injury to evaluate the regenerative effects of PBMC secretomes. Application via a commercially available hydrogel was fast and easy. The animals showed no clinical or laboratory signs of infection and the dressings were well tolerated.

The quality and stability of the newly regenerated skin are important after thermal injuries, as patients are prone to develop scar contractions, unstable scars with recurring ulcerations, and have a higher risk of malignant neoplasms^49^. The development of a fully differentiated epidermis is important to prevent the penetration of pathogens, as well as allergens that could sensitize the patient through a defective skin barrier^50^,^51^. We found a significantly increased thickness of the epidermal layer together with an increased number and length of rete ridges at the epidermal-dermal junction zone in the wounds treated with Apo-Sec^PBMC^. The hyperplastic epidermal layer at the edge of regenerating skin wounds is an essential component of the migrating cell pool that initiates rapid closure of the wound gap. The migration of keratinocytes then leads to further steps in the wound healing process^52^. Rete ridges are vertical protrusions of the epidermis into the dermal layer that increase the contact between the epidermal layer and the vessels in the dermal zone, providing it with more nutrients. In addition, rete ridges interlock the dermal-epidermal junction and render the skin less vulnerable to shear stress^53^. Our results reveal the formation of more stable epidermal-dermal junctions after the application of PBMC secretomes. These findings are of particular interest to surgeons treating burn patients, as split-thickness skin grafts are highly susceptible to shear stress the first few days after surgery. The increased number of rete ridges can also be interpreted as a result of improved epidermal regeneration and proliferation leading to faster protrusion of the epidermal layer into the dermis. Flattened rete ridges are a common finding in solar lentigo^54^. Another issue for burn victims is the high rate of infection after trauma due to the defective or missing skin barrier. Therefore, we investigated the rate of keratinocyte-differentiation in the epidermis after topical application of Sec^PBMC^ or Apo-Sec^PBMC^. After staining for keratin-10, we found a fully differentiated epidermis in wounds treated with PBMC secretomes, suggesting the formation of a complete and functional skin barrier. Wounds treated with control substances were only in the early stages of epidermal differentiation. However, we did not observe faster re-epithelialization in wounds treated with PBMC secretomes. One possible reason for this observation is that we used only young and healthy animals in this study. Therefore, wound healing was a priori expected to be fast, leading to smaller differences between the treatment and control groups. Another reason is that the mesh ratio of 3:1 that we used may be too small to observe a clinically significant difference in re-epithelialization rates after 10 days. We chose this model because it most closely resembles the clinical setting in large-scale burn injuries. Interestingly, the rate of re-epithelialization was similar in the wounds treated with the medium control alone. The exact underlying mechanisms however, have to be determined. We found no significant difference between the groups regarding the extent of open wound area on day 10. The slightly increased value in the Apo-Sec^PBMC^ group may be attributable to the trend towards less wound contraction in this group, thus increasing the total wound area on day 10.

The formation of new blood vessels is crucial for successful skin grafting. Skin transplants are completely separated from the circulation and initially rely on diffusion for nutritious support. The survival of the graft ultimately depends on the ingrowth of newly formed blood vessels, which occurs as early as day 3 after transplantation^55^. We found a markedly increased number of CD31+ and ASMA+ cells in the dermal layer of wounds treated with Apo-Sec^PBMC^ at the clinically relevant time point 5 days after skin grafting. These data suggest a pro-angiogenic effect of topically applying apoptotic PBMC secretome. Our findings are corroborated by the increased numbers of CD31+ cells after application of PBMC secretomes in a previous murine model of acute wound healing^18^. As the secretomes used in these experiments contain a plethora of different pro-angiogenic factors, the observed effects may not be attributable to a single factor, but rather to the natural mixture of paracrine factors that were applied, resembling a more physiological composition. In a previous study we selectively blocked different factors, including IL-8, VEGF, and MMP-9, but were not able to attenuate the observed effects^33^. We did not find a significant difference between the wound treatments regarding ICG measurements. This observation was possibly due to the already increased perfusion of the wound area after trauma and surgery, where the additional effect of the treatment may be too marginal to be captured, or to the insensitivity of the measurement method. ICG measurement may not be the method of choice for the evaluation of perfusion in this model as fibrin deposition and granulation tissue can interfere with the result as the depth of penetration into the tissue amounts only to some millimetres^56^. Further studies are needed to show if these findings of increased markers of blood vessels can also lead to a functional improvement of wound perfusion. Split-thickness skin grafting is an extremely reliable and effective method of wound coverage in healthy patients, leaving little room for improvement. However, co-morbidities such as diabetes, wound infections, and oedema are associated with a decrease in graft take rates and angiogenesis^57^. The wound biopsies taken for histological analysis included areas with open wounds and areas with skin grafts. In this study, we did not differentiate between these areas regarding angiogenesis. Future studies are planned to investigate in detail the effects of PBMC secretomes in chronic, non-healing wounds without the use of skin grafts.

Macroscopically, we observed a trend towards less wound contraction in wounds treated with Apo-Sec^PBMC^. Functionally, wounds treated with paracrine factors from apoptotic cells exhibited improved – yet without a significant difference – biomechanical properties, i.e., increased laxity, energy absorption, elastic deformation, and less stiffness. These short-term results indicate a possible beneficial effect of Apo-Sec^PBMC^ on skin regeneration and scar quality after thermal injuries and skin grafting. A soft and elastic scar and minimal contraction is highly desirable after split-thickness skin grafting. Excessive scar formation and contraction lead to functional impairment and bad aesthetic results^58^. One possible mechanism for the improved scarring described in this study is the decreased number of mast cells in the wounds treated with PBMC secretomes. Chronic activation and high numbers of mast cells are found in hypertrophic scars and keloids, forms of pathological scarring, whereas fewer mast cells are present in regular scars^59^,^60^. Cytokines released by mast cells after trauma intensify and extend the inflammatory response during wound healing. Therefore, increased mast cells presence and activation may influence scarring and wound remodelling as activated mast cells remain in the scar up to one year after trauma^61^. Thus far, our finding of a diminished mast cell population may represent a surrogate marker of better scarring after secretome application. The exact mechanisms responsible for these effects remain to be elucidated and warrant further studies.

In conclusion, we describe the positive effects of paracrine factors derived from apoptotic human PBMCs cultures during wound healing *in vivo*. We observed improved epidermal regeneration and differentiation, a trend towards better scar quality, and increased numbers of CD31+ and ASMA+ cells as markers for angiogenesis in a porcine full-thickness burn and skin grafting model. The cell-free, lyophilized PBMC secretomes can be obtained easily from healthy volunteers and can be stored as a ready-to-use agent in the clinical setting. Compared to therapies using isolated stem cells or progenitor cells, the cell-free secretome therapy described in this study comprises several advantages. Unlike freshly prepared cells, the freeze-dried secretomes can be stored for long time periods. They can be used off-the-shelf when needed and may also be produced for a patient as a prophylactic measure for future use. In addition, the possible heterologous use of the secretome therapy renders the fast and immediate treatment – e.g. for the treatment of burn wounds – possible. This is not the case for stem cell therapies as the isolation and purification of autologous cells requires the right infrastructure and adequate timing. The secretomes used in this study contain the complete physiologic composition of the factors secreted by PBMCs. The observed effects are therefore not the results of a single factor but rather attributable to the multitude of proteins, lipids, and extracellular vesicles that are released into the medium under cell culture conditions. Here we evaluated the effects of topically applied secretomes in an animal model that closely resembles the clinical setting of burn injury and early skin grafting. Therefore, the used *in vivo* model in this study was not solely designed to prove faster wound healing but to show additional effects of the secretome therapy when applied together with the currently established treatment options.

A limitation of this study is the lack of long-term follow-up observations to show the effects on scarring and functional outcome. Scar quality and scar contracture are important factors after burn injuries. The results in this study describe early changes in postoperative scarring. In order to evaluate the effects of secretome therapy on definite scarring, observations over longer time periods are needed. Another limitation is the relatively low number of animals included in the study. Despite the fact that we included only young and healthy pigs we still observed significant improvements in wound healing. Further studies are needed to evaluate the effect of Sec^PBMC^ and Apo-Sec^PBMC^ on chronic, non-healing wounds due to co-morbidities. The use and evaluation of human PBMC-derived secretomes aims to support the future treatment of human patients. We therefore chose the human compound in our study. This may be a limitation of the study design as possible cross-species effects are not excluded from the results.

The Austrian Federal Office for Safety in Health Care has approved the GMP production site for autologous and allogeneic Apo-Sec^PBMC^ and endorsed clinical trials including topical and systemic *in vivo* application. Allogeneic Apo-Sec^PBMC^ was categorized by the regulatory bodies as a “biological” and further clinical trials have to meet the standards required by conventional drug development. The data presented in this manuscript were the basis for a Phase I clinical trial utilizing the topical application of autologous Apo-Sec^PBMC^ to investigate the safety and tolerability of this new drug compound in male subjects with artificial dermal wounds (ClinicalTrial.gov Identifier: NCT02284360).

## Methods

### Preparation of PBMC secretomes and control substances

Twenty-one buffy coats from healthy volunteers were purchased from the Austrian Red Cross and used to acquire PBMCs. Blood draws were performed at the Austrian Red Cross after obtaining informed consent from all volunteers. The experimental protocol was approved by the ethics committee of the Medical University of Vienna (vote 2010/034). All experiments were performed in accordance with the Good Scientific Practice guidelines of the Medical University of Vienna and all relevant guidelines and regulations. PBMCs were isolated from heparinized whole blood by Ficoll-Paque density gradient centrifugation at 800 g for 15 min. (GE Healthcare Bio-Sciences AB, Uppsala, Sweden) The resulting layer of mononuclear cells was carefully transferred to a new centrifugation tube and washed twice in Hank´s balanced salt solution (HBSS). The resulting PBMCs were incubated for 24 hours at a concentration of 25 × 10^6^ cells in serum-free CellGro culture medium (CellGenix, Freiburg, Germany). No antibiotics were added. Incubation was performed in a standard cell culture incubator at 37 °C with 5% CO_2_ and 95% relative humidity. Immediately prior to incubation, PBMCs used for the production of the secretome from apoptotic PBMCs (Apo-Sec^PBMC^) were subjected to gamma irradiation (60 Gray). This irradiation step induced apoptosis in the majority of PBMCs over a period of 24 hours^34^. Beer *et al*. found that 58% of PBMCs were annexin V-fluorescein/propidium iodide positive at 20 hours after irradiation^27^. PBMCs used for the production of the secretome from living PBMCs (Sec^PBMC^) were cultured without prior irradiation. After an incubation period of 24 hours, the cell culture supernatant was harvested and subjected to centrifugation (268 g, 9 min) to remove cell debris and the supernatants pooled. The medium control (medium) was treated identically without the addition of cells. The supernatants were sterile filtered (Whatman Filter 0.2 μm, GE Healthcare, Little Chalfont, UK), divided into aliquots, and lyophilized to produce a dry powder. The powder was subsequently stored at −80 °C.

### Topical application using hydrogel as the carrier substance

The secretome of 5 × 10^7^ cells was used for the treatment of one wound. To create the desired concentration, the secretome of 3 × 10^8^ cells was dissolved in 3 ml of normal saline solution (NaCl, B. Braun, Melsungen, Germany). This stock solution was mixed with 15 ml of hydrogel (NuGel, Systagenix, Gatwick, West Sussex, UK) by gentle shaking. This preparation step was performed immediately prior to application. A total of 3 ml of the final solution was evenly applied to each wound using a sterile spatula. This volume was sufficient to cover the entire wound area. Medium controls were added in equal amounts. In the NaCl control group (NaCl), only normal saline solution without cell culture medium was added to the hydrogel.

### Animals, anaesthesia, and peri-operative care

Animal experiments were approved by the ethics committee of the University of Kaposvar, Hungary (vote: 23.1/02322/009/2008). All experiments were performed in accordance with relevant guidelines and regulations. Six female DanBred pigs weighing approximately 30 kg with an approximate age of 12 weeks were housed individually at the Animal Resource Facility. The animals were fasted overnight prior to performing the experiments. A detailed time line of the procedures and evaluations is provided in Fig. 1a. A previously described porcine model of standardized contact burns was used^37^. Briefly, anaesthesia was induced by intramuscular injection of 6 mg/kg ketamine hydrochloride and 2 mg/kg xylazine. After intubation, the pigs were weighed, clipped, and depilated, and then placed on an operating table in the prone position. A venous catheter was placed in an ear vein. Isoflurane was used to maintain anaesthesia and intra-operative analgesia achieved using sufentanil. Heart rate and oxygen saturation were monitored throughout the surgical procedure by a qualified veterinarian. Fluid resuscitation was administered using Ringer’s solution (B. Braun). Postoperative analgesia was provided with transdermal fentanyl patches (75 μg/h). Perioperative and postoperative antimicrobial prophylaxis was administered as 1 g cefazolin i.v. and 500 mg levofloxacin p.o. for 7 days. The animals had free access to food and water.

### Creation of standardized burn injuries

Contact burns were created paravertebrally under aseptic conditions by placing a heated aluminium bar (custom-made at the Ludwig Boltzmann Institute for Experimental and Clinical Traumatology, AUVA Research Center, Vienna, Austria) on the dorsum of the animal. The aluminium bar was heated to 200 °C using a Meeker gas burner. The core temperature of the bar was monitored with a digital thermometer. The heated bar was placed on the animal at the pre-determined spot for 30 seconds. The application pressure was measured with a 50 ml syringe attached to the aluminium bar via a heat insulation unit. This device was designed to exert a pressure of 0.4 kg/cm^2^ when the piston was pushed into the barrel of the syringe with one hand (from the 20 ml to the 10 ml mark), while the other hand held the heat insulation unit to prevent any additional pressure on the heat transfer bar (Fig. 1b). These parameters were previously shown to induce a full-thickness burn injury^37^. Burn sites of approximately 40 cm^2^ were created on the dorsum of the animal with a distance of 4 cm between each site and from the spine. The total burn size did not exceed 10% of the entire body surface area. A separate burn wound was created for each of the four treatment and control groups (NaCl, medium, Sec^PBMC^, and Apo-Sec^PBMC^).

### Excision, autologous skin grafting, and application of therapy

To simulate the routine clinical setting of full-thickness thermal injuries and subsequent surgical treatment, the necrotic tissue was excised to the level of the underlying muscular fascia 24 hours after the initial burn. For autologous skin harvesting, the distal dorsum and hind quarters of the animal were used. Split-thickness skin grafts (0.5-mm-thick) were harvested from two separate donor sites using a commercially available, compressed-air-driven dermatome (Zimmer, Warsaw, IN, USA), meshed at a 3:1 ratio, and fixed to the wound with skin staples (Covidien, Dublin, Ireland). Immediately after skin grafting, Sec^PBMC^, Apo-Sec^PBMC^, or control substances (medium and NaCl) were applied topically using hydrogel as the carrier substance. The allocation of therapies or controls to the respective fields was random. Each animal was treated with all controls and therapies. This procedure and the dressing changes were performed under general anaesthesia. Dressings were applied using non-sticky silicone oil-emulsion gauze (Jelonet®, Smith&Nephew, London, UK). The gauze was fixed using transparent, double polyurethane film (Opsite®, Smith&Nephew, London, UK). The dressings were further fixed and immobilized using elastic bandage (VetRap®, 3 MHealth Care, St. Paul, MN, USA), taking care not to impair the animal’s breathing or movement. The last dressing layer consisted of Goat tube® (Sullivan Supplies, Houston, TX, USA).

### Dressing changes and laboratory parameter profiles

The therapies or controls were re-applied during the dressing changes on postoperative days 2 and 5. On day 10, the dressings were removed and the animals euthanized after assessing the wounds. Blood draws were performed before and after thermal injury and during the dressing changes. Routine laboratory parameters (haemoglobin, white blood cell count) were determined by the central laboratory of the University of Kaposvar. Serum levels of IL-1b, IL-6, and TNF-alpha were determined using commercially available porcine-specific ELISA kits (R&D Systems, Minneapolis, MN, USA).

### Macroscopic wound measurements and planimetry

Two standardized digital photographs were taken of each wound by the same photographer. A metal ruler was placed at one edge of the picture to allow quantitative comparisons of wound sizes. The photographs were analysed by two blinded observers using ImageJ software^62^. The total wound size and the open wound areas (border zone, open spaces in the mesh graft, dislocation of the skin graft, and zones of non-adherence) were quantitatively measured to calculate the open wound area on days 0 and 10. The wound contraction rate was calculated as the difference between total wound size on days 0 and 10.

### Clinical assessment of wounds

The wounds were assessed clinically according to a standardized scheme using the scale adapted from Branski *et al*.^7^. During every dressing change, the following parameters were evaluated by the same blinded observer: graft dislocation (0: no dislocation, 1: partial dislocation, 2: full dislocation) and graft adherence (0: no adherence, 1: tissue partly viable, 2: tissue fully viable and adherent). The amount of visible granulation tissue, the degree of re-epithelialization (1: 0–20% of wound area, 2: 20–40%, 3: 40–60%, 4: 60–80%, 5: 80–100%), and fibrin deposition (1: 0–20% of wound area, 2: 20–40%, 3: 40–60%, 4: 60–80%, 5: 80–100%) were also determined.

### Histology

Wound biopsies were taken from the outer zones of the wound area at a distance of approximately 1 cm to the wound edge. Biopsies were taken from the same spot for all fields. A different spot was chosen for the repeated biopsies (Supplementary Fig. S4). The sample collection was performed during dressing changes using a standard 6 mm disposable biopsy punch (Kai Medical, Solingen, Germany). On day 10, additional excision biopsies were taken from the wound edges, including both parts of the surrounding healthy skin and the wound area, in order to directly compare wound tissue and healthy skin (Supplementary Fig. S4). Tissues were fixed in formalin for a minimum of 24 hours and embedded in paraffin. Standard H&E staining was performed on 5-μm-thick paraffin sections. The mean thickness of the newly formed epidermal layer in the wounded section was evaluated on postoperative day 10 by measuring the area of the epidermal layer and dividing it by the length of the external border. Using this method, the mean thickness of the total length was calculated. The extent and number of rete ridges was measured semi-quantitatively by two blinded observers (0: no rete ridges, 1: incipient formation of rete ridges/little depth, 2: few rete ridges/intermediate depth, 3: intermediate number of rete ridges/pronounced depth, 4: many rete ridges/very pronounced depth). In addition, the ratio between the length of the inner and outer epidermal border was calculated to quantify the extent of rete ridges.

### Immunohistochemistry

Angiogenesis (anti-CD31), mature blood vessels (alpha smooth muscle Actin, ASMA), epidermal differentiation (anti-keratin 10), and mast cell prevalence (anti-mast cell tryptase) were analysed by immunohistochemistry. Staining was performed on paraffin-embedded tissues after antigen retrieval by boiling in citrate-buffer (pH = 6, Dako, Glostrup, Denmark) in a microwave for 5 min. After blocking the sections with 10% normal goat serum for 1 hour, the slides were incubated overnight in a humidified chamber at 4 °C with either an anti-keratin-10 (Covance, Berkeley, CA, USA), anti-CD31 (Spring Biosciences, Pleasanton, CA, USA), anti-mast-cell tryptase (Abcam, Cambridge, UK), anti-ASMA (Abcam), or isotype-matched control (Abcam) antibody diluted in PBS containing 2% bovine serum albumin (BSA) and 10% goat serum. To visualize the stainings, sections were incubated with a horseradish peroxidase-linked secondary antibody in PBS containing 2% BSA and 10% normal goat serum for 1 h, followed by incubation with DAB Chromogen tablets (Dako). After washing, nuclear staining was performed by incubation with haematoxylin for 10 sec. Slides were mounted with Fluoprep (bioMérieux, Marcy l’Etoile, France). CD31+ and ASMA+ cells were quantitatively analysed by tissue cytometry using HistoQuest™ software (TissueGnostics, Vienna, Austria). Cells were counted in four fields per slide. Two fields from the superficial dermal layer and two fields from the deep dermal layer were evaluated. The pictures were taken randomly by a blinded observer. Mast cells were counted in four fields per slide by a blinded observer. Keratin-10 sections were used to assess the quality and stage of differentiation of the newly formed epidermis.

### Indocyanine green perfusion measurements

Perfusion of the grafted wounds was measured using previously described ICG perfusion measurements on days 2, 5, and 10^63^. Under general anaesthesia and after removal of the dressing, pigs were placed in the prone position. The digital camera system including the near-infrared light source was placed centrally above the dorsum of the pig. ICG (ICG-Pulsion, Pulsion Medical Systems, Munich, Germany) was injected via a peripheral venous catheter at a concentration of 0.5 mg/kg, diluted in 10 ml of NaCl. The video recording started 10 seconds before injection of ICG and continued for 3 minutes after the injection. A patch indicating the positive control was placed at the center of the visual field. Wound perfusion was analysed using IC-VIEW software (Pulsion Medical Systems).

### Biomechanical analysis

The BTC-2000™ (Surgical Research Laboratory, Nashville, TN, USA) was used to test the biomechanical properties of the wounds on day 10^64^. A suction chamber with a diameter of 20 mm was used and measurements were performed in the central part of the wound. A maximum negative pressure of 150 mmHg was applied over 15 sec and the deformation of the skin was measured by a laser beam. Skin quality was assessed using the following parameters: ratio of elasticity to elastic deformation (elasticity%), high values represent more elastic skin; elastic deformation (in mm), the amount of skin displacement to maximum pressure; ratio of laxity to elastic deformation (laxity%), indicates slack or looseness; stiffness (in mmHg/mm), slope of the stress/strain curve used, includes size and shape, higher values indicate tighter skin; energy absorption (mmHg × mm), area under the stress/strain curve, indicates overall softness or compliance, high values indicate softer, more compliant skin.

### Statistical analysis

Statistical analyses were performed using IBM SPSS Statistics 20 software package (IBM, Armonk, NY, USA) and GraphPad Prism 5.0 software (GraphPad Software, La Jolla, CA, USA). Data are given as mean ± standard deviation if not otherwise stated. Group comparisons between treatment and control groups were performed using the unpaired Student’s t-test for metric variables or the non-parametric Mann-Whitney U test for all other variables. A P-value <0.05 was considered significant. P-values were corrected using the Holm–Bonferroni method.

## Additional Information

**How to cite this article**: Hacker, S. *et al*. Paracrine Factors from Irradiated Peripheral Blood Mononuclear Cells Improve Skin Regeneration and Angiogenesis in a Porcine Burn Model. *Sci. Rep*. **6**, 25168; doi: 10.1038/srep25168 (2016).

## Supplementary Material

Supplementary Information

## Figures and Tables

**Table 1:** Results of biomechanical wound measurements using the BTC-2000™ system are shown.

	NaCl		Medium		Sec^PBMC^		Apo-Sec^PBMC^	
	mean	SD	mean	SD	mean	SD	mean	SD
Laxity (%)	28.23	6.66	30.67	16.69	17.02	12.85	38.25	17.01
Elastic Deformation (mm)	1.87	0.54	1.85	0.33	1.76	0.40	2.14	0.43
Stiffness (mmHg)	93.58	28.17	88.34	12.83	90.46	12.73	78.91	18.02
Energy Absorption (mmHg x mm)	125.44	34.16	124.65	19.17	122.22	20.03	145.50	33.56
Elasticity (%)	43.18	13.83	40.62	9.23	46.33	26.96	39.20	7.83
