# Supplementary material for: Paracrine Factors from Irradiated Peripheral Blood Mononuclear Cells Improve Skin Regeneration and Angiogenesis in a Porcine Burn Model
Source: Sci Rep. 2016 Apr 29;6:25168. doi: 10.1038/srep25168 (PMC4850437; doi:10.1038/srep25168)
Supplement: Supplementary Information [file srep25168-s1.pdf]

Supplementary information for:

Paracrine Factors from Irradiated Peripheral Blood Mononuclear Cells Improve Skin Regeneration and Angiogenesis in a Porcine Burn Model

Authors: Stefan Hacker [1 2], Rainer Mittermayr [3], Stefanie Nickl [1], Thomas Haider [2 4], Diana Lebherz-Eichinger [2], Lucian Beer [2], Andreas Mitterbauer [2], Harald Leiss [5], Matthias Zimmermann [2], Thomas Schweiger [2], Claudia Keibl [3], Helmut Hofbauer [2], Christian Gabriel [6], Mariann Pavone-Gyöngyösi [7], Heinz Redl [3], Erwin Tschachler [8], Michael Mildner [8] *, Hendrik Jan Ankersmit [2 9] *

Affiliations:
[1] Division of Plastic and Reconstructive Surgery, Medical University of Vienna, Waehringer Guertel 18-20, 1090 Vienna, Austria
[2] Christian Doppler Laboratory for Cardiac and Thoracic Diagnosis and Regeneration, Waehringer Guertel 18-20, 1090 Vienna, Austria
[3] Ludwig Boltzmann Institute for Experimental and Clinical Traumatology, AUVA Research Center, Donaueschingenstraße 13, 1200 Vienna, Austria
[4] Department of Trauma Surgery, Medical University of Vienna, Waehringer Guertel 18-20, 1090 Vienna, Austria
[5] Division of Rheumatology, Medical University of Vienna, Waehringer Guertel 18-20, 1090 Vienna, Austria
[6] Red Cross Blood Transfusion Service of Upper Austria, Krankenhausstraße 7, 4017 Linz
[7] Division of Cardiology, Medical University of Vienna, Waehringer Guertel 18-20, 1090 Vienna, Austria
[8] Department of Dermatology, Medical University of Vienna, Waehringer Guertel 18-20, 1090 Vienna, Austria
[9] Division of Thoracic Surgery, Medical University of Vienna, Waehringer Guertel 18-20, 1090 Vienna, Austria
* corresponding authors MM and HJA contributed equally

Corresponding Authors:
Hendrik Jan Ankersmit & Michael Mildner
Divison of Thoracic Surgery & Department of Dermatology
Medical University of Vienna
Waehringer Guertel 18-20, 1090 Vienna
eMail: hendrik.ankersmit@meduniwien.ac.at
eMail: michael.mildner@meduniwien.ac.at
Phone : +43-1-40400-67770, Fax : -69770

Supplementary Figure S1

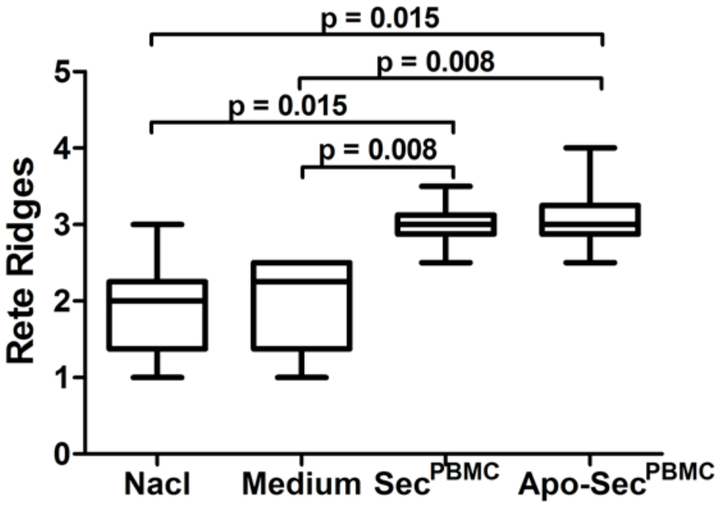

Supplementary Figure S1

A semi-quantitative analysis of the quality and the amount of rete ridges showed a significantly increased development of these structures in the treatment groups compared to the controls. n=6.

Supplementary Figure S2

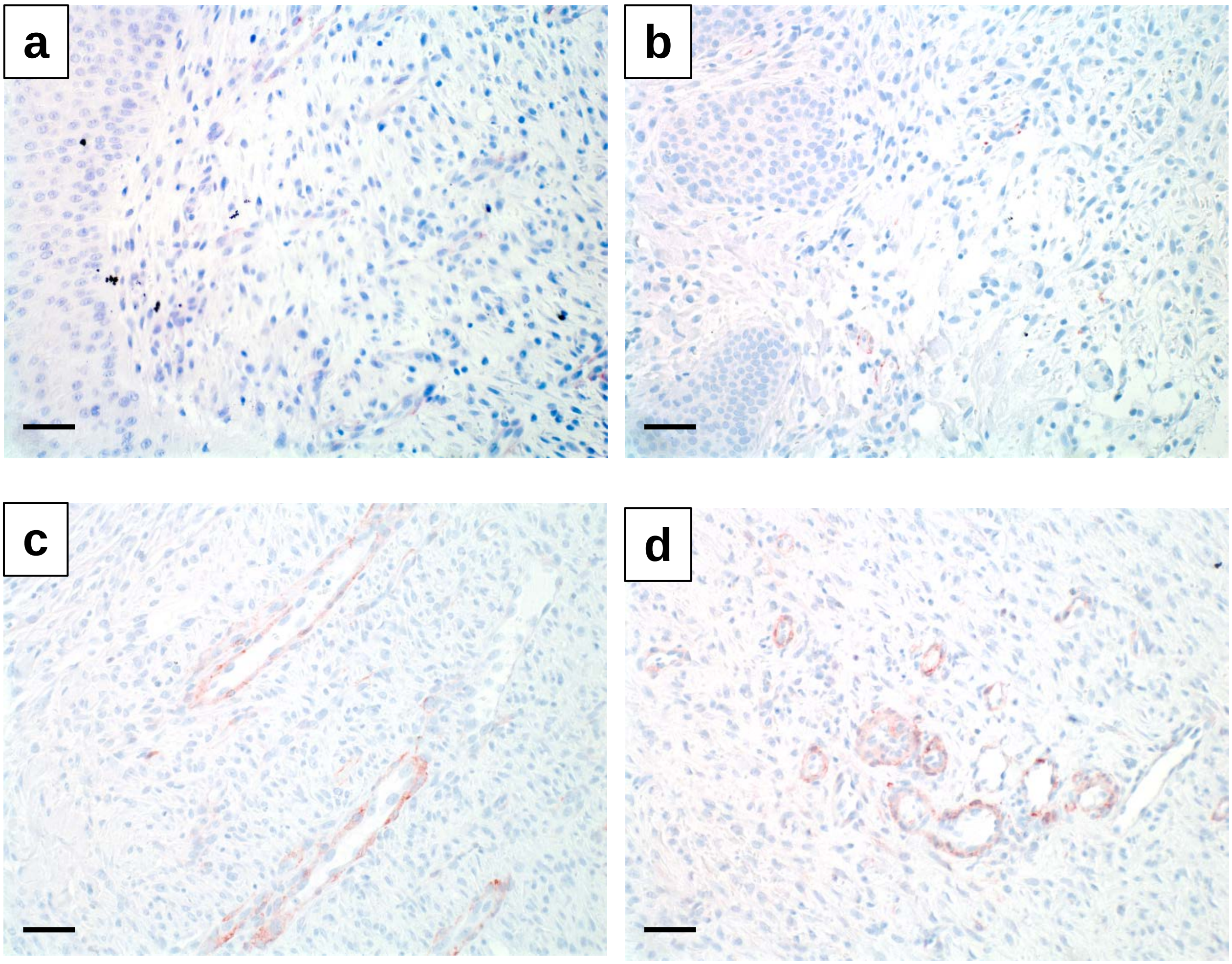

Supplementary Figure S2

Punch biopsy sections taken on day 5 were stained for ASMA. Representative samples of the NaCl (a), medium (b), Sec^{PBMC} (c), and Apo-Sec^{PBMC} (d) treated wounds are shown. 200x magnification, scale bar: 50µm. n=6.

Supplementary Figure S3

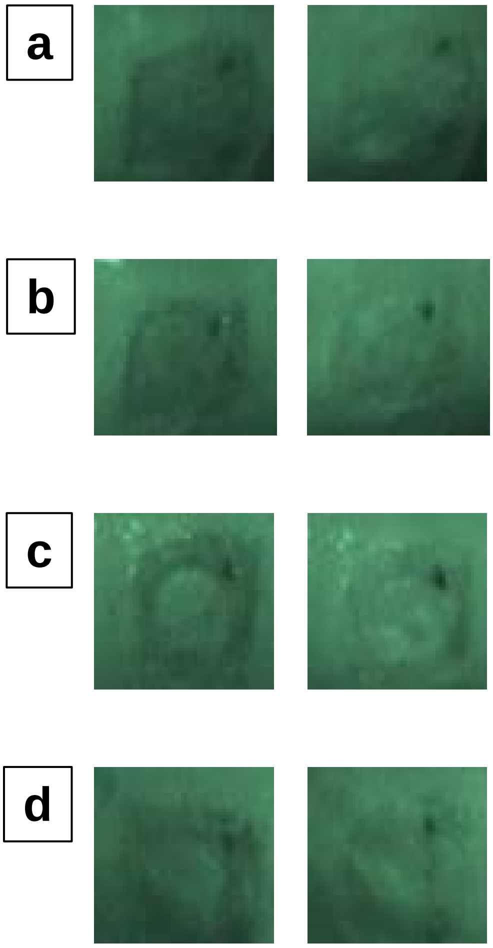

Supplementary Figure S3

ICG measurements were performed to evaluate the perfusion of the wounds. Representative samples on postoperative day 5 of the NaCl (a), medium (b), Sec^{PBMC} (c), and Apo-Sec^{PBMC} (d) treated wounds are shown. The images on the left side were taken before the application of ICG, the images on the right side were taken after the application.

Supplementary Figure S4

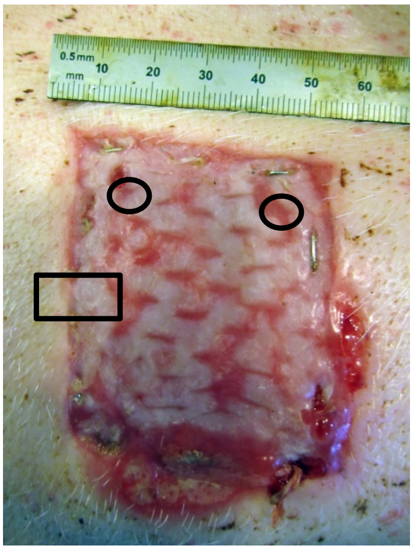

Supplementary Figure S4

Circles indicate the location of the biopsies taken on postoperative days 2 and 5. The rectangle shows the location of the excision biopsy performed on postoperative day 10.
